# P14 Fatal case of liver abscess caused by hypervirulent *Klebsiella pneumoniae* in an adolescent: clinical and laboratory study

**DOI:** 10.1093/jacamr/dlag102.020

**Published:** 2026-06-26

**Authors:** Yue Li, Zheng Li, Suyun Qian, Fang Dong, Qing Wang, Pengfei Zhang, Kaihu Yao

**Affiliations:** Beijing Children’s Hospital, Beijing, China; Beijing Children’s Hospital, Beijing, China; Beijing Children’s Hospital, Beijing, China; Beijing Children’s Hospital, Beijing, China; Beijing Children’s Hospital, Beijing, China; Beijing Children’s Hospital, Beijing, China; Beijing Children’s Hospital, Beijing, China

## Abstract

**Background:**

Hypervirulent variants of *Klebsiella pneumoniae* (hvKp) are able to cause life-threatening pyogenic liver abscess (PLA). There is an urgent need to raise our awareness of such cases in paediatric populations.

**Objectives:**

To report the clinical characteristics of hvKp caused fatal case of PLA complicated with bacteraemia in an adolescent, and to identify the microbiological and genomic features of the causative strain.

**Methods:**

Hypermucoviscosity phenotype of the strains was determined by string test. Virulence of the strains were measured by serum resistance assay and *G. mellonella* larvae killing assay. Antimicrobial susceptibility was determined by broth microdilution method. Genetic information was obtained by whole genome sequencing and bioinformatics analysis.

**Results:**

A 14-year-old boy with diabetes mellitus (DM) was admitted to our hospital with diagnosis of PLA complicated with bacteraemia. A hypermucoviscous hvKp strain KPN_19-106 was isolated from both drainage fluid within the liver abscess cavity and blood of the patient. KPN_19-106 belonged to ST380/K2 serotype, and exhibited stronger serum resistance and higher *in vivo* lethality capability than a well-characterized hvKp NTUH-K2044. Although KPN_19-106 was susceptible to most antibiotics, no sign of improvement was observed during treatment with susceptible antibiotics. Whole genome sequencing revealed that the isolate integrated multiple mobile genetic elements related to virulence.

**Conclusions:**

Antibiotics susceptible hvKp is able to cause fatal case of PLA complicated with bacteraemia in adolescents with no improvement during treatment with antibiotics. The causative strain integrated multiple virulence genes and exhibited higher virulence both *in vitro* and *in vivo* than NTUH-K2044.

